# Exploring potential circRNA biomarkers for cancers based on double-line heterogeneous graph representation learning

**DOI:** 10.1186/s12911-024-02564-6

**Published:** 2024-06-06

**Authors:** Yi Zhang, ZhenMei Wang, Hanyan Wei, Min Chen

**Affiliations:** 1 School of Computer Science and Engineering, Guilin University of Technology, Guilin, 541004 China; 2 Guangxi Key Laboratory of Embedded Technology and Intelligent System, Guilin University of Technology, Guilin, 541004 China; 3 School of Big Data, Guangxi Vocational and Technical College, Nanning, 530003 China; 4 Pharmacy School, Guilin Medical University, Guilin, 541004 China; 5 School of Computer Science and Technology, Hunan Institute of Technology, Hengyang, 421010 China

**Keywords:** Graph networks, Representation learning, Heterogeneous, Neural network, node2vec

## Abstract

**Background:**

Compared with the time-consuming and labor-intensive for biological validation in vitro or in vivo, the computational models can provide high-quality and purposeful candidates in an instant. Existing computational models face limitations in effectively utilizing sparse local structural information for accurate predictions in circRNA-disease associations. This study addresses this challenge with a proposed method, CDA-DGRL (Prediction of CircRNA-Disease Association based on Double-line Graph Representation Learning), which employs a deep learning framework leveraging graph networks and a dual-line representation model integrating graph node features.

**Method:**

CDA-DGRL comprises several key steps: initially, the integration of diverse biological information to compute integrated similarities among circRNAs and diseases, leading to the construction of a heterogeneous network specific to circRNA-disease associations. Subsequently, circRNA and disease node features are derived using sparse autoencoders. Thirdly, a graph convolutional neural network is employed to capture the local graph network structure by inputting the circRNA-disease heterogeneous network alongside node features. Fourthly, the utilization of node2vec facilitates depth-first sampling of the circRNA-disease heterogeneous network to grasp the global graph network structure, addressing issues associated with sparse raw data. Finally, the fusion of local and global graph network structures is inputted into an extra trees classifier to identify potential circRNA-disease associations.

**Results:**

The results, obtained through a rigorous five-fold cross-validation on the circR2Disease dataset, demonstrate the superiority of CDA-DGRL with an AUC value of 0.9866 and an AUPR value of 0.9897 compared to existing state-of-the-art models. Notably, the hyper-random tree classifier employed in this model outperforms other machine learning classifiers.

**Conclusion:**

Thus, CDA-DGRL stands as a promising methodology for reliably identifying circRNA-disease associations, offering potential avenues to alleviate the necessity for extensive traditional biological experiments. The source code and data for this study are available at https://github.com/zywait/CDA-DGRL.

## Introduction

Circular RNAs (circRNAs) are a new type of non-coding RNAs involved in the development of certain diseases, which plays an important role in gene expression and signaling pathways [1]. Compared with other non-coding RNAs, circRNAs as a biomarker of disease has demonstrated with better stability and integrity, thus offering great potential in tumor diagnosis [2, 3]. Gene expression and protein synthesis in cancer cells are also regulated by circRNAs [4]. Traditional works in biological validation for identifying the association between circRNA and disease are time-consuming and usually lack specificity, although with high prediction accuracy [5]. Meanwhile, biological databases coming from traditional biological experiments and related literature increasingly provide the convenience and basis for computational methods to identify circRNA-disease associations more efficiently and economically [6]. Currently, existing computational methods for predicting circRNA-disease associations are classified into two major categories broadly: network computing-based models and machine learning-based models.

### Network computing-based models

These models leverage circRNA (disease) similarity network and known circRNA-disease associations to construct the heterogeneity network. Subsequently, algorithms tailored for this network are employed to forecast potential associations. Lei et al. [7] proposed a method named RWRKNN, which integrated the random walk with restart (RWR) and k-nearest neighbors (KNN) to predict circRNA-disease associations. However, RWRKNN highly relies on priori information of circRNAs and diseases, it is slightly inadequate in revealing the relationship between isolated diseases and new circRNAs. Li et al. [8] proposed a novel method named DWNCPCDA based on DeepWalk and Network Consistency Projection. An important innovation of DWNCPCDA was adopted DeepWalk, an embedded method of network, to learn embedding of nodes in the network of known circRNA-disease associations. Zhang et al. [9] proposed a linear neighborhood label propagation method, named CD-LNLP, to predict circRNA-disease associations. CD-LNLP resulted in good performance mainly attributing to the following factors: the application of linear neighbor similarity (LNS) guaranteeing the basic effectiveness, and only using the known and reliable circRNA-disease associations as prior information. CD-LNLP also could not be applied in prediction of associations involving new circRNAs or isolated diseases.

### Machine learning-based models

These models utilize circRNA (disease) similarity network and known circRNA-disease associations to train supervised or unsupervised learning algorithms. These algorithms iteratively optimize their internal parameters to extract latent features from the circRNA and disease data. Lan et al. [10] proposed a new computational method (KGANCDA) to predict circRNA-disease associations based on knowledge graph attention network. CircRNA-disease knowledge graphs were constructed by collecting multiple relationship data between different types of nodes (circRNAs, diseases, miRNAs and lncRNAs). Embeddings of each entity in circRNA-disease knowledge graphs were obtained with attention network by distinguishing the importance of information from neighbors. Besides the low-order neighbor information, KGANCDA could also capture high-order neighbor information from multi-source associations to alleviate the problem of raw-data sparsity. Ma et al. [11] proposed a novel algorithm CRPGCN to predict circRNA-disease associations based on Graph Convolutional Network (GCN) constructed with Random Walk with Restart (RWR) and Principal Component Analysis (PCA). RWR was used to calculate similarity between nodes. After that, PCA that was used to reduce dimensions and extract features intensified the association of circRNAs with diseases. However, CRPGCN produced the biased results due to some data were isolated in the process of data fusion. Zheng et al. [12] introduced iCDA-CGR, a novel approach aimed at identifying circRNA-disease associations by leveraging Chaos Game Representation (CGR). By incorporating sequence information and quantifying nonlinear relationships, iCDA-CGR addressed the limitation of model coverage. Nevertheless, there remains a scope for enhancing the predictive accuracy of iCDA-CGR. Li et al. [13] proposed SIMCCDA, a method that leverages inductive matrix completion techniques to impute the missing values within the known circRNA-disease association matrix. This approach reformulates the association prediction task as a recommendation system problem, achieving good performance with reduced memory requirements and training time. However, SIMCCDA cannot be applied to the prediction of new diseases without any associations or isolated circRNAs. Zuo et al. [14] proposed DMCCDA, an association prediction method based on double matrix completion. DMCCDA employs matrix completion methods to reconstruct the known association matrix. Subsequently, it utilizes the reconstructed matrix alongside a corresponding Gaussian similarity matrix to create a combined matrix, which is again reconstructed using matrix completion. The final prediction score integrates the results from these steps. Despite its methodological novelty, DMCCDA exhibits limitations in performance compared to alternative methods.

In recent years, deep learning-based models have emerged as a powerful tool in bioinformatics [5]. These models represent biological systems as graphs, where nodes represent biological entities and edges represent interactions between them [15]. Graph representation learning, a technique within deep learning, extracts features from graph networks and learns low-dimensional representations of nodes, links, and subgraphs, preserving the graph's topology and intrinsic properties [16]. Several studies have employed graph representation learning for various biological association prediction tasks: Zhang et al. [17] proposed a computational model based on graph representation learning that was composed of GCN and graph factorization (GF), named iGRLCDA, to identify circRNA–disease associations. Peng et al. [18] proposed a novel end-to-end heterogeneous graph representation learning-based model, called EEG-DTI, to identify drug–target interactions. Zhao et al. [19] proposed a novel model, namely HINGRL, to predict drug-disease associations with graph representation learning on heterogeneous information network. Jiang et al. [20] presented a novel computational model combining sparse auto-encoder and rotation forest (SAEROF) to predict drug-disease association. Ha et al. [21] proposed a node2vec-based neural collaborative filter, named NCMD, to predict miRNA-disease associations. Zhao et al. [22] proposed a novel method to predict drug-target interactions based on large-scale graph representation learning. Zhao et al. [23] proposed MotifMDA, a novel motif-aware model that integrates high and low-order structural information for miRNA-disease association prediction.

Extra-tree classifiers have also proven effective in bioinformatics tasks due to their ability to introduce randomization and achieve good flexibility and accuracy [24, 25]. Extra-tree classifiers have been successfully applied in leukocyte classification [26], lncRNA-protein interactions identification [27], and cardiovascular disease prediction [28].

While several computational methods have been proposed, they exhibit shortcomings such as reliance on prior information, inability to accommodate new circRNAs or isolated diseases, biased results, and limited prediction accuracy [7, 9–12, 15]. Furthermore, the inherent complexity of extracting relevant features from heterogeneous graphs poses a substantial challenge to the development of robust models for circRNA-disease association prediction [20–22, 24, 25, 29, 30]. To overcome these challenges, we propose a novel approach termed CDA-DGRL (CircRNA-Disease Association Prediction via Double-Line Graph Representation Learning). This innovative model integrates diverse biological data sources, employs advanced feature extraction techniques, and comprehensively analyzes both local and global graph structures to enhance the identification of circRNA-disease associations. By addressing these challenges, CDA-DGRL aims to provide a more accurate and efficient means of predicting circRNA-disease associations, thereby facilitating advancements in disease diagnosis and treatment.

- Step 1, diverse biological information encompassing circRNA functional similarity, disease semantic similarity, circRNA (disease) Gaussian interaction profile kernel similarity, and circRNA-disease known associations were integrated to form integrated circRNA (disease) similarity. These integrated similarities were then utilized to construct the circRNA-disease heterogeneous network (CDHN).
- Step 2, the integrated circRNA (disease) similarity metric from step 1 was then fed into a sparse auto-encoder to extract node features for both circRNAs and diseases within the CDHN.
- Step 3, local graph networks were built by inputting the node features of CDHN into a GCN, enabling the capture of local graph structures.
- Step 4, global graph networks were constructed using node2vec, employing depth-first sampling within CDHN to comprehend the broader network structure comprehensively.
- Step 5, the combination of local and global graph networks was inputted into an extra-tree classifier to identify potential circRNA-disease associations.

CDA-DGRL represents a novel approach that leverages the strengths of both local and global graph structures. By integrating diverse biological data sources, employing a sparse auto-encoder for feature extraction, and comprehensively analyzing both the fine-grained relationships (local structures) and the broader network context (global structures) within the circRNA-disease heterogeneous network, CDA-DGRL effectively identifies circRNA-disease associations.

## Results

### Experiment dataset

From the circR2Disease database [31], we assembled a dataset comprising 739 experimentally validated associations, involving 661 circRNAs and 100 diseases. Following the removal of redundant entries, our focus narrowed to 650 non-repetitive associations linked specifically to human complex diseases as the known circRNA-disease associations. This refined benchmark dataset involved 585 distinct circRNAs and encompassed 88 unique complex diseases.

### Evaluation metric and method

When evaluating circRNA-disease node pairs, whose prediction scores surpassing a predefined threshold are classified as positive samples; otherwise, those falling below the threshold are labeled as negative samples. True positive rate (TPR) and false positive rate (FPR) were computed at various threshold values, generating multiple TPR and FPR groups. These data points were utilized to construct receiver operating characteristic (ROC) curves plotting TPR against FPR. Common evaluation metrics including area under the ROC curve (AUROC), area under the precision-recall (PR) curve (AUPR), accuracy, sensitivity, precision, specificity, and Matthews's correlation coefficient (MCC) were employed to evluate the predictive performance of the compared models under comparison. To mitigate the impact of result variance, a fivefold cross-validation method was iterated 10 times to ensure robustness. The average values derived from these repetitions were calculated to yield final evaluation results.

### Evaluation result and analyzation

#### Five-fold-cross-validation

After implementing fivefold cross-validation, the results for each evaluation metric obtained from CDA-DGRL are presented in Table 1.

**Table 1:** Evaluation Results on Each Fold

fold	accuracy	sensitivity	specificity	precision	MCC	AUROC	AUPR
0	0.9577	0.9462	0.9692	0.9685	0.9156	0.9848	0.9897
1	0.9577	0.9385	0.9769	0.9760	0.9161	0.9872	0.9900
2	0.9538	0.9538	0.9538	0.9538	0.9077	0.9878	0.9905
3	0.9577	0.9308	0.9846	0.9837	0.9167	0.9852	0.9882
4	0.9577	0.9615	0.9538	0.9542	0.9154	0.9904	0.9923
**mean**	**0.9569 ± 0.0017**	**0.9462 ± 0.0121**	**0.9677 ± 0.0138**	**0.9672 ± 0.1323**	**0.9143 ± 0.0037**	**0.9866 ± 0.0022**	**0.9897 ± 0.0014**

Based on the outcomes detailed in Table 1 for each metric, CDA-DGRL exhibited notable predictive performance across all folds within the fivefold cross-validation. The consistent results observed across different folds underscore the model's proficiency and stability, affirming CDA-DGRL's capability for both excellent performance and consistent reliability.

#### Ablation experiment

To better assess the impact and significance of incorporating different network structures on addressing data sparsity within the biological network, we conducted ablation experiments employing three distinct experimental schemes: ① local graph structure only; ② global graph structure only; ③ both local and global graph structures. Subsequent to performing fivefold cross-validation, the detailed experimental outcomes are presented in Table 2.

**Table 2:** Results of Different Scheme Settings

sheme	accuracy	sensitivity	specificity	precision	MCC	AUROC	AUPR
①	0.8708 ± 0.0140	0.8585 ± 0.0359	0.8831 ± 0.0138	0.8803 ± 0.0096	0.7424 ± 0.0271	0.9275 ± 0.0165	0.9434 ± 0.0056
②	0.9370 ± 0.0064	0.9339 ± 0.0117	0.9400 ± 0.0064	0.9397 ± 0.0061	0.8739 ± 0.0128	0.9768 ± 0.0038	0.9822 ± 0.0026
**③(ours)**	**0.9569 ± 0.0017**	**0.9462 ± 0.0121**	**0.9677 ± 0.0138**	**0.9672 ± 0.1323**	**0.9143 ± 0.0037**	**0.9866 ± 0.0022**	**0.9897 ± 0.0014**

The outcomes in Table 2 illustrate that the third experimental scheme (ours) achieved the best predictive performance across all evaluation metrics. The first scheme only utilizes the local network structure, focusing on the immediate relationships between circRNAs and diseases. While this approach can capture fine-grained details about these relationships, it may miss broader network context that could be informative for prediction. The second scheme solely leverages the global network structure, analyzing the overall connectivity patterns within the network. This can capture the broader context of circRNA and disease interactions but may lack the specificity of local relationships. For instance, it might identify circRNAs with similar disease associations even if they lack direct functional similarity. The third experimental scheme (ours) integrates both local and global network structures. This allows the model to capture both fine-grained relationships between circRNAs and diseases and the broader network context. The superior performance of our scheme supports the theoretical notion that combining local and global network structures allows the model to extract more comprehensive features, leading to more accurate circRNA-disease association prediction.

#### Classifier comparison

To comprehensively validate our model, we employed various classifiers, such as random forest (RF) [17], logistic regression (LR) [32], K-nearest neighbor classifier (KNN) [7], Gaussian Parsimonious Bayes (Gaussian NB) [17], and extra-tree classifier (ET). Each classifier was individually incorporated into our model to assess their respective contributions toward achieving optimal predictive performance. Employing fivefold cross-validation with default parameters, we meticulously evaluated the performance of each classifier. Detailed evaluation results are presented in Table 3, outlining their respective predictive capacities.

**Table 3:** Performance of Different Classifiers

classifier	accuracy	sensitivity	specificity	precision	MCC	AUROC	AUPR
RF	0.9354 ± 0.0069	0.9400 ± 0.0126	0.9308 ± 0.0109	0.9315 ± 0.0099	0.8710 ± 0.0138	0.9801 ± 0.0044	0.9842 ± 0.0033
LR	0.7231 ± 0.0380	0.7538 ± 0.0696	0.6923 ± 0.02495	0.7095 ± 0.0263	0.4483 ± 0.0787	0.7617 ± 0.0330	0.7154 ± 0.0390
KNN	0.8300 ± 0.1260	0.9539 ± 0.0196	0.7062 ± 0.0263	0.7648 ± 0.0151	0.6817 ± 0.0250	0.9269 ± 0.0068	0.9299 ± 0.0073
Guassian NB	0.6785 ± 0.0307	0.6523 ± 0.0503	0.7046 ± 0.0409	0.6886 ± 0.0319	0.3580 ± 0.0610	0.7459 ± 0.0351	0.7610 ± 0.0337
**ET(ours)**	**0.9569 ± 0.0017**	**0.9462 ± 0.0121**	**0.9677 ± 0.0138**	**0.9672 ± 0.1323**	**0.9143 ± 0.0037**	**0.9866 ± 0.0022**	**0.9897 ± 0.0014**

The analysis of Table 3 reveals that the integration of the extra-tree classifier (ET) resulted in superior performance metrics compared to other classifiers. Specifically, the ET implementation facilitated an improvement of 0.65%, 22.49%, 5.97%, and 24.07% in AUROC values over alternative classifiers. Furthermore, the utilization of ET within our model led to the achievement of the highest AUPR value, showcasing enhancements of 0.55%, 27.43%, 5.98%, and 22.87% compared to other classifiers, respectively.

#### Model comparison

**Fig. 1:**
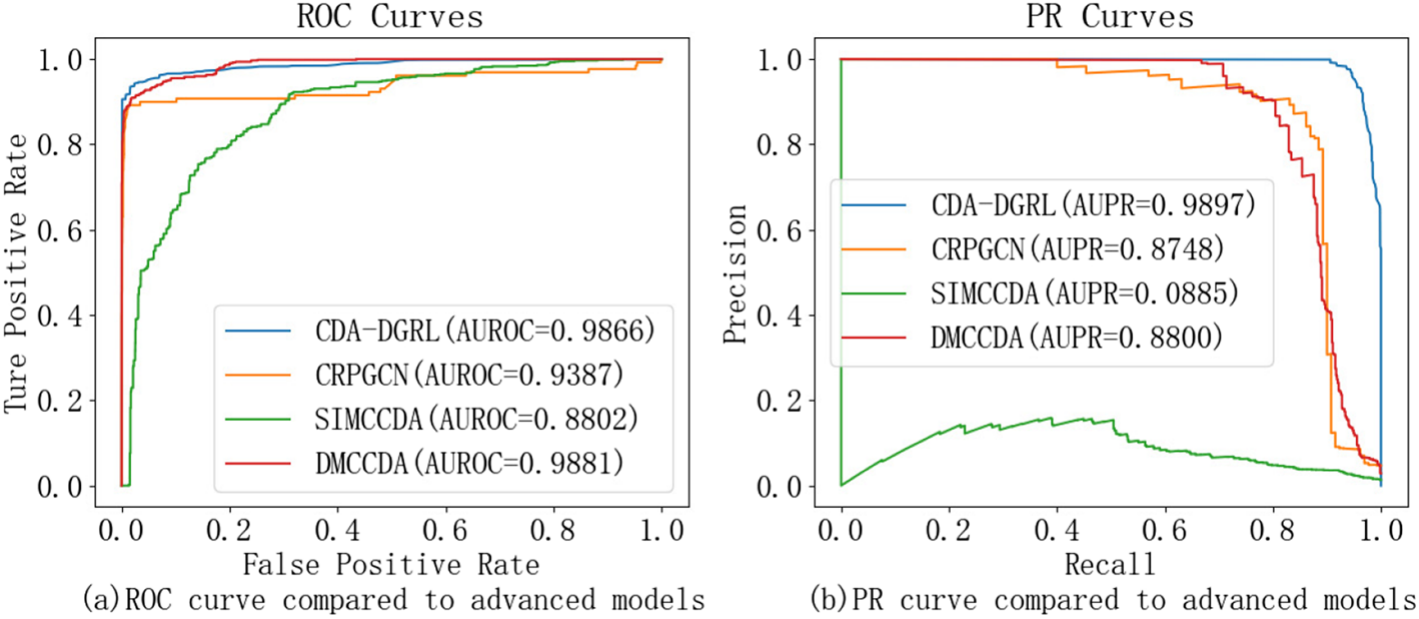
AUROC and AUPR curves for models engaged in comparison

To assess the effectiveness of our CDA-DGRL model, we conducted a comparative analysis against three related state-of-the-art models, SIMCCDA [13], CRPGCN [11] and DMCCDA [14]. This comparison was conducted using the refined benchmark dataset outlined in Sect. "Experiment Dataset". Hyperparameter selection for all involved models was guided by relevant lectures to ensure optimal configuration. Following a rigorous fivefold cross-validation process, comprehensive evaluation results are visually presented in Table 4 and Fig. 1.

**Table 4:** Performance of Model for Comparison

model	accuracy	sensitivity	specificity	precision	MCC	AUROC	AUPR
SIMCCDAA	0.8317	0.7708	0.9965	0.0556	0.1772	0.8802	0.0885
CRPGCN	0.9696	0.6077	0.9988	0.9634	0.7567	0.9387	0.8748
DMCCDA	0.9224	0.0948	0.9993	0.1346	0.3412	0.9881	0.8800
**CDA-DGRL (ours)**	**0.9569 ± 0.0017**	**0.9462 ± 0.0121**	**0.9677 ± 0.0138**	**0.9672 ± 0.1323**	**0.9143 ± 0.0037**	**0.9866 ± 0.0022**	**0.9897 ± 0.0014**

As the results shown in Table 4, our CDA-DGRL performs excellently across most key metrics, showing a balanced performance advantage. While it may not be the best in some individual metrics, its overall performance is very strong. Notably, it excels in accuracy, sensitivity, MCC, and AUC. While CDA-DGRL is slightly inferior in certain individual metrics compared to DMCCDA and CRPGCN, its overall performance is more balanced. For example, CDA-DGRL performs exceptionally well in sensitivity, precision, AUROC, and AUPR, indicating its potential advantage in handling imbalanced datasets and practical applications. As depicted in Fig. 1, CDA-DGRL demonstrates superior performance in both AUROC and AUPR values, especially on imbalanced datasets. Although DMCCDA achieves a marginally higher AUROC value (0.25%) than our CDA-DGRL, its AUPR value is notably lower by 10.97% in comparison. While SIMCCDA solely relies on network similarity for prediction, CDA-DGRL integrates diverse biological data sources and leverages both local and global network structures. This comprehensive approach likely contributes to CDA-DGRL's advantage in capturing complex relationships between circRNAs and diseases. Compared to CRPGCN, which utilizes GCNs to learn features from the local network structure, CDA-DGRL additionally analyzes the broader network context. This theoretically allows CDA-DGRL to capture more informative features, leading to its superior performance. Interestingly, DMCCDA achieves a marginally higher AUROC value than CDA-DGRL. However, its AUPR value is notably lower. DMCCDA incorporates multi-source information but may not explicitly capture fine-grained relationships between circRNAs and diseases, potentially explaining the lower AUPR. Conversely, CDA-DGRL's focus on both local and global structures likely contributes to its strong performance in both metrics. Consequently, CDA-DGRL exhibits the most comprehensive and superior performance across both evaluation metrics, highlighting the effectiveness of our proposed double-line graph representation learning approach for circRNA-disease association prediction.

#### Robustness verification

**Fig. 2:**
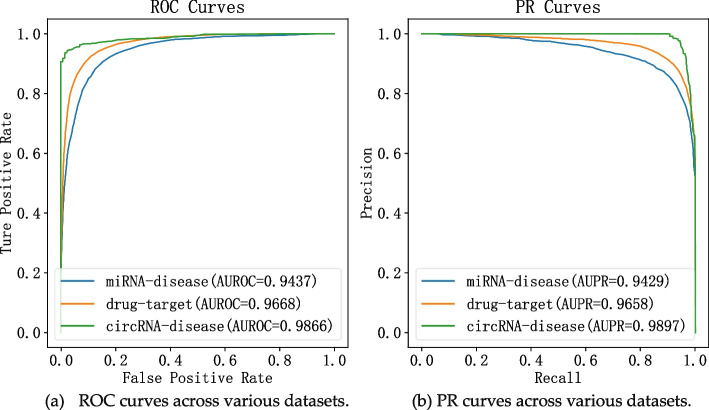
Results for robustness verification. **a** ROC curves across various datasets. **b** PR curves across various datasets

Additional experiments were conducted to verify the robustness of our model across various domains: circRNA-disease association prediction, miRNA-disease association prediction, and drug-target interaction prediction. The dataset concerning circRNA-disease association was sourced from the previously described benchmark dataset. Subsequently, datasets for miRNA-disease association and drug-target interaction were acquired and processed in accordance with methodologies outlined in literature [33] and literature [22], respectively. The miRNA-disease association dataset encompasses 5430 established associations involving 495 distinct miRNAs and 383 diseases. On the other hand, the drug-target interaction dataset consists of 11,396 known associations involving 984 drugs and 635 proteins. Employing a rigorous five-fold cross-validation process, ROC plots and PR plots were generated for the three datasets, as depicted in Fig. 2. These experiments were conducted with the objective of assessing our model's predictive performance and robustness across diverse molecular interaction domains. They serve to demonstrate the efficacy of our model in predicting circRNA-disease associations, miRNA-disease associations, and drug-target interactions, showcasing its versatility and effectiveness.

As depicted in Fig. 2, CDA-DGRL attained AUC values of 0.9437, 0.9668, and 0.9866, along with AUPR values of 0.9429, 0.9658, and 0.9897 for circRNA-disease association data, miRNA-disease association data, and drug-target interaction data, respectively. These experimental outcomes substantiate the model's applicability across datasets characterized by distinct scales and content compositions. Furthermore, the results underscore its robustness and notable generalization capacity.

### Case study

Many researchers are trying hard to minimize the incidence of cancers. Global cancer statistics [34] reported that breast cancer is the most prevalent type of cancer in women worldwide and ranks second in terms of death tolls. For gastric cancer, the five-year survival rate is generally 5–25%. Among the cancers, gastric cancer is more deadly [35]. To validate the predictive capabilities of CDA-DGRL in real-world scenarios, this study conducted case studies focusing on breast cancer and gastric cancer. Through computational analyses, the model identified circRNAs associated with these two cancers. After sorting the resultant association prediction scores in descending order, the top 10 ranked circRNAs related to each case were selected to be validated with cross-referencing relevant literature and reports available in the PMID database. The detailed results are presented in Tables 5 and 6 as follows.

**Table 5:** Top 10 breast cancer-related candidate circRNAs

rank	circRNA	PMID
1	circHIPK3(also known as hsa_circRNA_100782 and hsa_circ_0000284)	34,135,597
2	ciRS-7(also known as CDR1as and hsa_circ_0001946)	31,245,927
3	circPVT1(also known as hsa_circ_0001821)	33,223,849
4	cir-ITCH (also known as hsa_circ_0001141 and hsa_circ_001763)	33,544,410
5	circCCDC66(also known as hsa_circ_00013130)	8,249,903
6	circPRKCI(also known as hsa_circ_0067934)	35,236,829
7	circ-Foxo3(also known as hsa_circ_0006404)	28,278,047
8	hsa_circRNA_103110(also known as hsa_circ_103110 and hsa_circ_0004771)	30,979,827
9	hsa_circ_0001649	unconfirmed
10	hsa_circ_0000064	unconfirmed

**Table 6:** Top 10 gastric cancer-related candidate circRNAs

rank	circRNA	PMID
1	hsa_circ_0001649	28,167,847
2	ciRS-7(also known as CDR1as and hsa_circ_0001946)	34,221,006
3	cir-ITCH(also known as hsa_circ_0001141 and hsa_circ_001763)	33,060,778
4	circCCDC66(also known as hsa_circ_0001313)	32,253,030
5	hsa_circ_0007534	unconfirmed
6	circPRKCI(also known as hsa_circ_0067934)	35,113,408
7	hsa_circ_0014717	28,544,609
8	circ-MCTP1(also known as hsa_circ_0005540)	unconfirmed
9	circHIPK3(also known as hsa_circRNA_100782 and hsa_circ_0000284)	33,680,975
10	circSMARCA5(also known as hsa_circ_0001445)	30,956,729

In Tables 5 and 6, both only two out of ten circRNAs predicted haven’t been found to have any evidence described in the literature of PubMed database. Alrough there is no direct description of the association between “hsa_circ_0001649” and breast cancer in the literature so far, literatue [36] studied the relationship between hsa_circ_0001649 and miR-20a and the underlying molecular mechanisms, and literature [37] demostrated the role for miR-20a in the regulation of breast cancer angiogenesis. An accompanying file on the Royal Society of Chemistry's website delineates the association between “hsa_circ_0000064” and breast cancer, despite the absence of a direct explicit description of this association within available literature. In Table 5, there's no direct description in any literature currently available that associates “hsa_circ_0007534” with gastric cancer. However, numerous pieces of literature demonstrate a direct association between "hsa_circ_0007534" and colorectal cancer as well as pancreatic cancer, both of which belong to cancers affecting parts of the digestive system [38–40]. We believe that forthcoming research will unveil evidence linking 'hsa_circ_0007534' to gastric cancer, a digestive system-related cancer. As for “circ-MCTP1”, another circRNA lacking direct evidence, it has been demonstrated to be associated with multiple system atrophy (MSA) [41]. Furthermore, it's noteworthy that all patients diagnosed with MSA exhibit gastrointestinal abnormalities [42]. The potential for discovering evidence linking "hsa_circ_0007534" to gastric cancer remains open for future exploration.

## Discussion

The precise identification of the association between circRNAs and diseases holds significant promise in expediting drug development, personalized diagnostics, and the treatment landscape for a spectrum of human diseases. In this study, we introduce a novel deep learning framework termed CDA-DGRL, which leverages a graph network structure and employs bilinear representation based on graph node features. This framework could capture both local and global structural information inherent in heterogeneous networks. By doing so, it mitigates the challenge of poor prediction accuracy stemming from the inherent sparsity of biological data. Notably, the model exhibits robustness and applicability across datasets with varying scales and contents. Our future endeavors involve the integration of diverse biological information, encompassing miRNA, lncRNA, and other pertinent elements, to construct an expansive circRNA-disease heterogeneity network. This holistic approach aims to enrich the pool of circRNA and disease-related information, facilitating more precise predictions of the association between circRNAs and diseases. With unraveling and interpreting the deep sea of circRNAs, it may serve as prognostic, diagnostic, and even therapeutic tools, or molecules to be targeted for biomedical research and clinical applications. While CDA-DGRL demonstrates promising performance, there is an opportunity to potentially enhance the effectiveness of local network structure representation. Inspired by the work presented in [43], we will explore how alternative attribute graph network construction methods might improve the model's capability to capture intricate rel.

## Materials and methods

### Network construction

#### CircRNA-Disease Heterogeneous Network (CDHN)

Utilizing the previously referenced benchmark dataset, a circRNA-disease association network was constructed and denoted as ${\mathbf{A}} \in {\mathbb{R}}^{n \times m}$, where the variables $n$ and $m$ represent the number of circRNAs and diseases involved, respectively. In this network, if a circRNA $c_{i}$ has a known association with disease $d_{j}$, the matrix element ${\mathbf{A}}(c_{i} ,d_{j} ) = 1$; conversely, ${\mathbf{A}}(c_{i} ,d_{j} ) = 0$. Subsequently, a heterogeneous network CDHN, represented by an adjacent matrix ${\mathbf{X}} \in {\mathbb{R}}^{(n + m) \times (n + m)}$, was constructed using the association information as follows:

1 $${\mathbf{X}} = \left[ {\begin{array}{*{20}c} 0 & {\mathbf{A}} \\ {{\mathbf{A}}^{T} } & 0 \\ \end{array} } \right]$$

where ${\mathbf{A}}^{T}$ represents the corresponding transpose matrix of ${\mathbf{A}}$. This construction results in a comprehensive heterogeneous network capturing both circRNA-disease associations and their interrelations.

#### Disease semantic similarity network

Semantic information regarding diseases was obtained from the U.S. National Library of Medicine database (https://www.nlm.nih.gov/mesh/), with which semantic similarities for diseases were calculated by using directed acyclic graphs (DAG) [44]. Within this framework, a disease node $d$ is represented by $DAG_{d} = \left( {d,T_{d} ,E_{d} } \right)$, where $T_{d}$ denotes the set encompassing all ancestors of disease $d$ (including $d$ itself), and $E_{d}$ signifies the set of edges connecting those diseases in the set. Consequently, the semantic contribution value of any disease $d$ to disease $d_{i}$ was defined with $SC_{{d_{i} }} \left( d \right)$:

2 $$S_{d_{i}} (d) = \left\{\begin{array}{cl} 1, & if\ d = d_{i}\\ \max \left\{ \gamma \times SC_{d_{i}} (d^{\prime}) | d^{\prime} \in child\ of\ d \right\}, & if\ d \ne d_{i} \end{array} \right.$$

where $\gamma$ represents the semantic contribution factor, empirically set to 0.5 in accordance with literature [44]. This formulation aims to quantify the semantic relationship between diseases based on their shared ancestry within the DAG framework.

The semantic value of disease $d_{i}$ is represented by $SV\left( {d_{i} } \right)$, with definition as:

3 $$SV\left( {d_{i} } \right) = \sum\limits_{{d \in T_{d} }} {SC_{{d_{i} }} \left( d \right)}$$

The matrix element within the disease semantic similarity network (denoted as ${\mathbf{DS}} \in {\mathbb{R}}^{m \times m}$) that represent the semantic similarity between disease $d_{i}$ and disease $d_{j}$ is denoted by ${\mathbf{DS}}\left( {d_{i} ,d_{j} } \right)$, with calculation as:

4 $${\mathbf{DS}}\left( {d_{i} ,d_{j} } \right) = \frac{{\sum\nolimits_{{d_{k} \in T_{{d_{i} }} \cap T_{{d_{j} }} }} {\left( {SC_{{d_{i} }} \left( {d_{k} } \right) + SC_{{d_{j} }} \left( {d_{k} } \right)} \right)} }}{{SV\left( {d_{i} } \right) + SV\left( {d_{j} } \right)}}$$

#### CircRNA functional similarity network

In accordance with the hypothesis suggesting that similar circRNAs tend to be associated with similar diseases and vice versa [45], circRNA functional similarity was calculated by integrating disease semantic similarity and experimentally validated circRNA-disease associations. The calculation involved determining the maximum semantic similarity value for any disease $d$ within the disease set $T = \left\{ {d_{1} ,d_{2} , \cdots ,d_{m} } \right\}$ was calculated as:

5 $$\max \left( {d,T} \right) = \mathop {\max }\limits_{1 \le i \le m} \left( {{\mathbf{DS}}\left( {d,d_{i} } \right)} \right)$$

Matrix ${\mathbf{FS}} \in {\mathbb{R}}^{n \times n}$ denotes the circRNA functional similarity network whose element ${\mathbf{FS}}\left( {c_{i} ,c_{j} } \right)$ represents the circRNA functional similarity between circRNA $c_{i}$ and circRNA $c_{j}$:

6 $${\mathbf{FS}}\left( {c_{i} ,c_{j} } \right) = \frac{{\sum\nolimits_{1 \le p \le r} {\max \left( {d_{p} ,T_{i} } \right) + \sum\nolimits_{1 \le q \le k} {\max \left( {d_{q} ,T_{j} } \right)} } }}{r + l}$$

where $T_{i}$ represents the set of diseases associated with circRNA $c_{i}$, $T_{j}$ represents the set of diseases associated with circRNA $c_{j}$, $r$ and $l$ denote the number of diseases in sets $T_{i}$ and $T_{j}$, respectively.

#### Gaussian interaction profile kernel similarity network

The sparsity inherent in the original circRNA-disease association network significantly impacts prediciton accuracy. To address this limitation, we introduced the Gaussian interaction profiles kernel similarity to fill the missing values within the original circRNA-disease association network [45]. Matrix ${\mathbf{CK}} \in {\mathbb{R}}^{n \times n}$ represents the Gaussian interaction profile kernel similarity for circRNAs, where the matrix element ${\mathbf{CK}}\left( {c_{i} ,c_{j} } \right)$ denotes the Gaussian interaction profile kernel similarity between circRNA $c_{i}$ and circRNA $c_{j}$:

7 $${\mathbf{CK}}\left( {c_{i} ,c_{j} } \right) = \exp \left( { - \lambda_{{\text{c}}} \left\| {{\mathbf{A}}\left( {c_{i} ,d_{j} } \right) - {\mathbf{A}}\left( {c_{j} ,d_{j} } \right)} \right\|^{2} } \right)$$

where the parameter $\lambda_{{\text{c}}}$ represents the control kernel bandwidth, employed to regulate the size of ${\mathbf{CK}}\left( {c_{i} ,c_{j} } \right)$:

8 $$\lambda_{{\text{c}}} = \frac{n}{{\sum\limits_{i = 1}^{n} {\left\| {{\mathbf{A}}\left( {c_{i} ,d_{j} } \right)} \right\|^{2} } }}$$

Similarly, the Gaussian interaction profile kernel similarity for diseases (${\mathbf{DK}} \in {\mathbb{R}}^{m \times m}$), wherein the matrix element ${\mathbf{DK}}\left( {d_{i} ,d_{j} } \right)$ undergoes a similar calcuation processes as above.

#### Integrated similarity network

To improve the relatively low accuracy caused by sparsity within the circRNA (disease) semantic similarity network, we combined circRNA (disease) Gaussian interaction profile kernel similarity with circRNA functional similarity (disease semantic similarity). This combination resulted in the formation of the integrated circRNA similarity network (${\mathbf{X}}_{c} \in {\mathbb{R}}^{n \times n}$) and the integrated disease similarity network (${\mathbf{X}}_{d} \in {\mathbb{R}}^{m \times m}$), respectively:

9 $${\mathbf{X}}_{c} \left( {c_{i} ,c_{j} } \right) = \left\{ {\begin{array}{*{20}c} {{\mathbf{FS}}\left( {c_{i} ,c_{j} } \right),} \\ {{\mathbf{CK}}\left( {c_{i} ,c_{j} } \right),} \\ \end{array} \begin{array}{*{20}c} {} & {\begin{array}{*{20}c} {if \, {\mathbf{FS}}\left( {c_{i} ,c_{j} } \right) \ne 0} \\ \end{array} } \\ {} & {if \, {\mathbf{FS}}\left( {c_{i} ,c_{j} } \right) = 0} \\ \end{array} } \right.$$

10 $${\mathbf{X}}_{d} \left( {d_{i} ,d_{j} } \right) = \left\{ {\begin{array}{*{20}c} {{\mathbf{DS}}\left( {d_{i} ,d_{j} } \right),} \\ {{\mathbf{DK}}\left( {d_{i} ,d_{j} } \right),} \\ \end{array} } \right.\begin{array}{*{20}c} {} & {\begin{array}{*{20}c} {if \, {\mathbf{DS}}\left( {d_{i} ,d_{j} } \right) \ne 0} \\ \end{array} } \\ {} & {if \, {\mathbf{DS}}\left( {d_{i} ,d_{j} } \right) = 0} \\ \end{array}$$

### Feature extraction

The relationships among nodes within HCDN are complex, and individual node features typically encompass multiple attributes. To precisely comprehend these relationships, node features necessitate extraction from various perspectives and dimensions to comprehensively capture the network's complexity.

#### Dimensionality reduction

The sparse auto-encoder could not only fix the redundancy and sparsity problems existing in the original benchmark dataset, but also enhance the model's generalization ability, mitigating overfitting during the training phase [20]. To reduce the dimensionality of the integrated circRNA (disease) similarity and obtain a more concise representation, a novel sparse auto-encoder based on a three-layer neural network structure was designed.

Integrated circRNA similarity network (${\mathbf{X}}_{c}$) as input was fed into the sparse auto-encoder. The optimal number of neurons in the hidden layer, minimizing data loss during the transformation from the original space (input layer) to the new feature space (output layer), was denoted by $k$, with a value set to 64 [22]. The input was compressed within the hidden layer, calculated as:

11 $${\vec{\mathbf{y}}}_{c} = \sigma \left( {{\vec{\mathbf{x}}}_{c} {\mathbf{W}}_{1} + {\vec{\mathbf{b}}}_{1} } \right)$$

where ${\vec{\mathbf{y}}}_{c} \in {\mathbb{R}}^{1 \times k}$, a vector within matrix ${\mathbf{Y}}_{c} \in {\mathbb{R}}^{n \times k}$, represents the encoded mapping outcome derived from the output layer. Matrix ${\mathbf{W}}_{1} \in {\mathbb{R}}^{n \times k}$ denotes the weight matrix from the input layer to the hidden layer, while ${\vec{\mathbf{x}}}_{c} \in {\mathbb{R}}^{1 \times n}$ denotes a vector within matrix ${\mathbf{X}}_{c}$. Vector ${\vec{\mathbf{b}}}_{1} \in {\mathbb{R}}^{1 \times k}$ represents the bias, and $\sigma (\cdot)$ denotes the activation function of the neurons.

Subsequently, within the output layer, ${\mathbf{Y}}_{c}$ was decompressed to reconstruct circRNA integration similarity (${\mathbf{X}}_{c}$), with calculation as:

12 $${\vec{\mathbf{z}}}_{c} = \sigma \left( {{\vec{\mathbf{y}}}_{c} {\mathbf{W}}_{2} + {\vec{\mathbf{b}}}_{2} } \right)$$

where ${\vec{\mathbf{z}}}_{c} \in {\mathbb{R}}^{1 \times k}$, a vector within matrix ${\mathbf{Z}}_{c} \in {\mathbb{R}}^{n \times k}$, represents the reconstructed outcome subsequent to the decompression. Matrix ${\mathbf{W}}_{2} \in {\mathbb{R}}^{k \times k}$ denotes the weight matrix from the hidden layer to the output layer, and vector ${\vec{\mathbf{b}}}_{2} \in {\mathbb{R}}^{1 \times k}$ represents the bias.

Throughout the aforementioned calculation processes, the dimensionality of integrated circRNA similarity underwent reduction, potentially resulting in the loss of circRNA-related information. To mitigate this loss, the sparse auto-encoder was trained by iteratively minimizing the loss between ${\mathbf{W}}_{1}$ and ${\mathbf{W}}_{2}$. Employing the gradient descent algorithm [19] to alternately optimize both the weight matrix and bias. Consequently, the loss function characterizing CDA-DGRL is defined as:

13 $$Loss = \frac{1}{n}\sum\limits_{i = 1}^{n} {\left\| {{\vec{\mathbf{y}}}_{c} - {\vec{\mathbf{z}}}_{c} } \right\|^{2} }$$

Similarly, the reconstruction of integrated disease similarity network ${\mathbf{X}}_{d}$ (denoted as ${\mathbf{Z}}_{d} \in {\mathbb{R}}^{m \times k}$) followed a parallel calculation process as the aforementioned steps. Subsequently, by concatenating ${\mathbf{Z}}_{c} \in {\mathbb{R}}^{n \times k}$ and ${\mathbf{Z}}_{d} \in {\mathbb{R}}^{m \times k}$ together, the final circRNA-disease feature matrix ${\mathbf{Q}} = \left[ {{\mathbf{Z}}_{c} ,{\mathbf{Z}}_{d} } \right]^{T} \in {\mathbb{R}}^{{\left( {n + m} \right) \times k}}$ was derived.

#### Local graph network structure

GCN is a semi-supervised technique that translates the topological relationships within a graph into topological graphs [22]. Through convolutional operations, GCN can acquire the embedding representation of nodes in the graph, enabling the direct extraction of structural information and node attributes. A spatial methodology employing a two-layer GCN configuration was used to capture the local structural details within the heterogeneous network HCDN:

14 $${\mathbf{H}}_{l} = {\text{ReLU}} \left( {{\tilde{\mathbf{D}}}^{{ - \frac{1}{2}}} {\mathbf{\tilde{X}\tilde{D}}}^{{ - \frac{1}{2}}} {\mathbf{WQ}}} \right)$$

15 $${\tilde{\mathbf{X}}} = {\mathbf{X}} + {\mathbf{I}}$$

where ${\mathbf{I}} \in {\mathbb{R}}^{{\left( {\text{n + m}} \right) \times \left( {\text{n + m}} \right)}}$ represents the identity matrix of matrix ${\mathbf{X}} \in {\mathbb{R}}^{(n + m) \times (n + m)}$, ${\tilde{\mathbf{D}}}$ signifies the metric matrix of ${\tilde{\mathbf{X}}}$, ${\mathbf{W}} \in {\mathbb{R}}^{{\left( {n + m} \right) \times \left( {n + m} \right)}}$ denotes the weight matrix initialized randomly for the network, ${\text{ReLU}} \left( \cdot \right)$ denotes the activation function utilized, and ${\mathbf{H}}_{l} \in {\mathbb{R}}^{{\left( {n + m} \right) \times k}}$ denotes the captured local graph network structure.

#### Global graph network structure

Node2vec is one type of graph representations that designs a flexible biased random walk technique. Node2vec generates traversal paths by integrating breadth-first (BF) sampling and depth-first (DF) sampling, introducing two hyperparameters $p$ and $q$, to smoothly transition between these two sampling methodologies [15, 46]. The adaptable biased random walk technique employed in Node2vec aims to preserve the high-order node proximities, thereby maximizing the network coverage while mapping nodes into a lower-dimensional feature space for learning node embeddings. For example, node $v$ denotes the current node, and the probability of visiting the subsequent node $x$, could be calculated as:

16 $$P\left( {t_{i + 1} = x|t_{i} = v} \right) = \left\{\begin{array}{cl} \frac{\pi_{vx}}{Z}, & if\ (v,x) \in E\\ 0, & otherwise \end{array} \right.$$

where $Z$ represents a normalizing constant, $\left( {v,x} \right) \in E$ denotes the existence of an edge connecting node $v$ and node $x$. When the current walk reaches node $v$ through the edge connecting node $t$ and node $v$, $\pi_{vx}$ denotes the unnormalized transition probability:

17 $$\pi_{vx} = \alpha_{pq} (t,x)w_{vx}$$

18 $$\alpha_{pq} (t,x) = \left\{ {\begin{array}{*{20}c} {\frac{1}{p}, \, if \, d_{tx} = 0} \\ {1, \, if \, d_{tx} = 1} \\ {\frac{1}{q}, \, if \, d_{tx} = 2} \\ \end{array} } \right.$$

where $w_{vx}$ represents the weight of the edge connecting node $v$ and node $x$, while $d_{tx}$ represents the shortest distance from node $t$ to node $x$. Utilizing formula (18), the global graph network structure of the heterogeneous network (${\mathbf{X}}$) was captured and is denoted by ${\mathbf{H}}_{g} \in {\mathbb{R}}^{{\left( {n + m} \right) \times k}}$. Following multiple rounds of experimentation, the optimal values for the hyperparameters $p$ and $q$ were set to 1.0 and 0.25, respectively. 

### Extra-tree classifier prediction

The local graph network structure ${\mathbf{H}}_{l} \in {\mathbb{R}}^{{\left( {n + m} \right) \times k}}$, and the global graph network structure ${\mathbf{H}}_{g} \in {\mathbb{R}}^{{\left( {n + m} \right) \times k}}$, were contacted together to derive an integrated network structure ${\mathbf{H}} \in {\mathbb{R}}^{{\left( {n + m} \right) \times 2k}}$:

19 $${\mathbf{H}} = {\mathbf{H}}_{l} ||{\mathbf{H}}_{g}$$

**Fig. 3:**
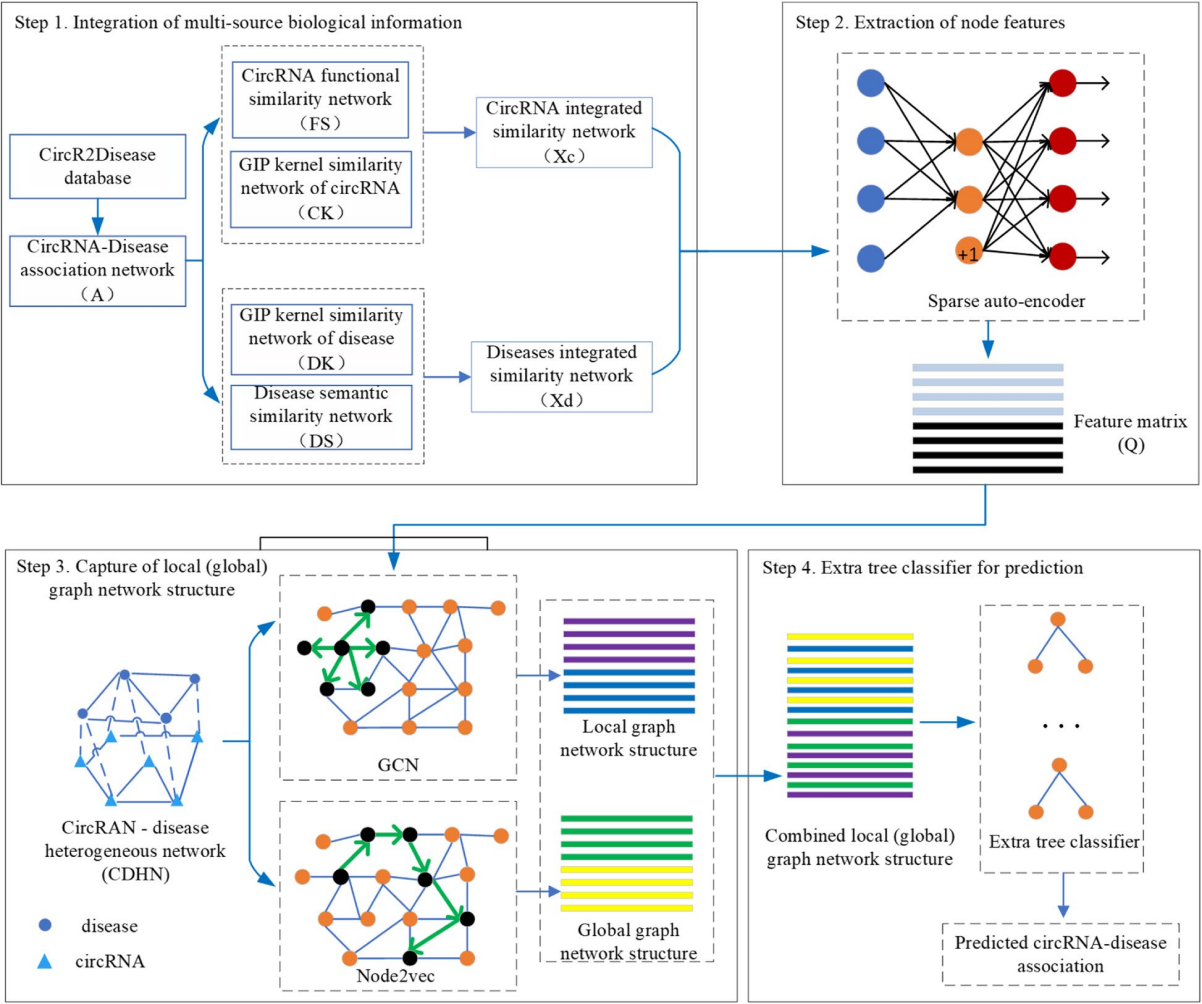
Model schematic depiction

Finally, matrix ${\mathbf{H}}$ was fed into the extra-tree classifier [24, 25] with utilizing default parameters for training purposes. This process yielded prediction scores representing circRNA-disease associations as the outputs. Therefore, the comprehensive workflow of our model, CDA-DGRL, is concisely illustrated in Fig. 3.

## Data Availability

Data is provided within the manuscript.
